# Notch-regulated miR-223 targets the aryl hydrocarbon receptor pathway and increases cytokine production in macrophages from rheumatoid arthritis patients

**DOI:** 10.1038/srep20223

**Published:** 2016-02-03

**Authors:** Jesús Ogando, Manuel Tardáguila, Andrea Díaz-Alderete, Alicia Usategui, Vanessa Miranda-Ramos, Dannys Jorge Martínez-Herrera, Lorena de la Fuente, María J. García-León, María C. Moreno, Sara Escudero, Juan D. Cañete, María L. Toribio, Ildefonso Cases, Alberto Pascual-Montano, José Luis Pablos, Santos Mañes

**Affiliations:** 1 Department of Immunology and Oncology, Centro Nacional de Biotecnología/CSIC, Madrid; 2 Servicio de Reumatología, Instituto de Investigación Hospital 12 de Octubre, Madrid; 3 Bioinformatics Unit, Centro Nacional de Biotecnología/CSIC, Madrid; 4 Centro de Investigación Príncipe Felipe, Valencia; 5 Centro de Biología Molecular Severo Ochoa/CSIC-UAM, Madrid; 6 Flow Cytometry Unit, Centro Nacional de Biotecnología/CSIC, Madrid; 7 Unitat d’Artritis, Hospital Clínic de Barcelona and Institut d’Investigacions Biomèdiques August Pí i Sunyer (IDIBAPS), Barcelona; 8 Institut de Medicina Predictiva i Personalitzada del Càncer, Badalona, Barcelona, Spain

## Abstract

Evidence links aryl hydrocarbon receptor (AHR) activation to rheumatoid arthritis (RA) pathogenesis, although results are inconsistent. AHR agonists inhibit pro-inflammatory cytokine expression in macrophages, pivotal cells in RA aetiopathogenesis, which hints at specific circuits that regulate the AHR pathway in RA macrophages. We compared microRNA (miR) expression in CD14^+^ cells from patients with active RA or with osteoarthritis (OA). Seven miR were downregulated and one (miR-223) upregulated in RA compared to OA cells. miR-223 upregulation correlated with reduced Notch3 and Notch effector expression in RA patients. Overexpression of the Notch-induced repressor HEY-1 and co-culture of healthy donor monocytes with Notch ligand-expressing cells showed direct Notch-mediated downregulation of miR-223. Bioinformatics predicted the AHR regulator ARNT (AHR nuclear translocator) as a miR-223 target. Pre-miR-223 overexpression silenced ARNT 3’UTR-driven reporter expression, reduced ARNT (but not AHR) protein levels and prevented AHR/ARNT-mediated inhibition of pro-inflammatory cytokine expression. miR-223 counteracted AHR/ARNT-induced Notch3 upregulation in monocytes. Levels of ARNT and of CYP1B1, an AHR/ARNT signalling effector, were reduced in RA compared to OA synovial tissue, which correlated with miR-223 levels. Our results associate Notch signalling to miR-223 downregulation in RA macrophages, and identify miR-223 as a negative regulator of the AHR/ARNT pathway through ARNT targeting.

Macrophages are an integral part of the innate immune system, with important functions in the onset and development of chronic inflammatory diseases^1^. Many pathological alterations in RA result from aberrant macrophage hyperactivation in the inflamed synovial membrane^2^. These activated macrophages are the main producers of pro-inflammatory factors, including tumor necrosis factor (TNF)-α, interleukin (IL)-1β and IL-6, targets of current therapies^3^. Synovial macrophage accumulation correlates well with disease activity and joint destruction, and is the most sensitive biomarker of a positive response to therapy^4^.

MicroRNA (miR), small noncoding RNA molecules of ~15–25 nucleotides, act as posttranscriptional negative regulators that fine-tune protein expression by binding to the 3′-untranslated regions (UTR) of their mRNA targets; this causes their degradation or translation repression depending on the degree of base-pair complementarity between the miR and its target mRNA^5^. Specific miR can also positively regulate gene expression^5^. miR participate in immune cell differentiation, maturation and function^6^, and their aberrant expression is associated with pathogenesis in several osteoarticular diseases. Synovial fibroblasts from RA patients upregulate miR-155, miR-203, miR-221, miR-222 and miR-323-3p; immune cells from these patients also show altered miR-16, miR-132, miR-146a, miR-155 and miR-223 levels^7^,^8^. The miR profile in RA patients differs from that in patients with OA^9^, a non-autoimmune disease characterised by low inflammation levels. The relevance of these miR in RA aetiopathogenesis is poorly understood.

The aryl hydrocarbon receptor (AHR), or dioxin receptor is a cytosolic sensor of xenobiotics such as benzo[*a*]pyrene (B*a*P), found in large amounts in tobacco smoke^10^, a known environmental risk factor for RA development and severity^11^. AHR also binds to endogenous ligands, including metabolites of arachidonic acid and tryptophan^12^. AHR-ligand complex translocates to the nucleus, where it forms a heterodimeric complex with the aryl hydrocarbon nuclear translocator (ARNT, also termed hypoxia-inducible factor-1β), which enables receptor binding to cognate DNA consensus sequences^12^ including xenobiotic metabolising enzymes of the cytochrome P450 superfamily members CYP1A1, CYP1A2 and CYP1B1 (ref. 10). A non-conventional, ARNT-independent pathway has also been reported^13^.

Evidence links AHR activation to RA pathogenesis. AHR agonists upregulate IL-1β in human synoviocytes^14^. AHR also contributes to the differentiation of T helper (Th)17 cells^15^, and is necessary for their IL-22 production^16^,^17^. AHR deficiency in T cells, but not in macrophages, inhibits development of experimental arthritis in mice^18^. In apparent contradiction, AHR enhances differentiation of regulatory T (Treg) cells^16^,^19^ and suppresses experimental autoimmune encephalomyelitis^20^. AHR stimulation also inhibits TNF-α, IL-6 and IL-1β expression in lipopolysaccharide (LPS)-stimulated macrophages^21^,^22^,^23^. These disparate results hint at cell type-specific circuits that regulate the AHR/ARNT pathway in specific contexts. In this study, we analysed the role of miR-223 as a part of these regulatory circuits in RA-derived macrophages.

## Results

### Macrophages from RA vs OA patients express distinct sets of miR

Given the effectiveness of current therapies, few RA patients now require replacement surgery, and availability of fresh RA synovial tissue is thus limited. In active RA, numerous neutrophils, lymphocytes and macrophages migrate from synovial tissue and accumulate in the synovial fluid (SF)^24^. CD14^+^ macrophage-like cells constitute the majority of inflammatory CD14^+^ cells isolated from SF^25^, and these cells display markers compatible with M1-skewed polarisation^26^. We analysed the miR expression profile in CD14^+^ cells isolated from SF of three RA patients with active disease. The CD14-based positive selection method rendered >95% CD14^+^CD3^−^ cell purity that showed no detectable IL-23 expression (not shown). As a reference, we used CD14^+^ cells isolated from the synovial membrane of three OA patients. OA is a low intensity inflammatory disease, and synovial fluid (SF) from OA patients has very low cellularity, which impedes the isolation of sufficient CD14^+^ cells to perform the analyses.

**Figure 1:**
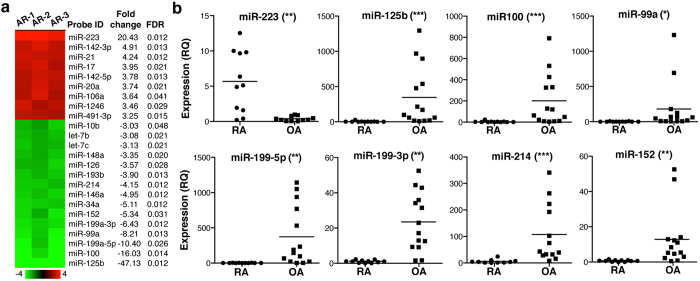
RA and OA patient macrophages express a specific miR profile. (**a**) Heat map showing the differentially expressed miR in CD14^+^ cells from OA and RA as determined by hybridisation in two-color microarrays. The x-fold change and FDR value are also indicated (*n* = 3 samples/condition). (**b**) miR validated by RT-qPCR in an independent set of 11 RA and 14 OA samples. Values are indicated as relative quantification (RQ), using as reference the sample with the lowest 2^−ΔΔCt^ value (RQ = 1). *p < 0.05, **p < 0.01, ***p < 0.001, Mann-Whitney *U*-test.

Our analysis identified 24 differentially regulated miR (≥3-fold), with a false discovery rate (FDR) <0.05 (Fig. 1a). Some up- and downregulated miR belong to the same family and are transcribed in a single mRNA; this was the case of clusters miR-17–92 (comprising miR-17, miR-20a and miR-106a), miR-125 (miR-125b, miR-99a, miR-100 and let-7), miR-199 (miR-199a, miR-199b, miR-214) and miR-152/148 (miR-152 and miR-148a).

Sixteen miR (7 up- and 9 downregulated) were selected for further validation by real-time, quantitative PCR (RT-qPCR) in an independent set of CD14^+^ cells isolated from SF (RA patients) or synovial tissue (OA patients). miR-223 was the only miR validated as upregulated in RA- compared to OA-derived cells (Fig. 1b). The miR-17–92 cluster and miR-142-5p tended to be upregulated in RA macrophages, but values were not significant (Supplementary Fig. S1). Seven downregulated miR of the miR-125, miR-199 and miR-152 clusters were validated; miR-34a and miR-146a were not confirmed (Supplementary Fig. S1). We focussed further analysis on miR-223, as it was the only miR upregulated in RA-derived cells, given the importance of the targets identified by bioinformatics (see below) and due to its implication in RA pathogenesis^27^,^28^,^29^,^30^,^31^.

### The Notch pathway regulates miR-223 expression

**Figure 2:**
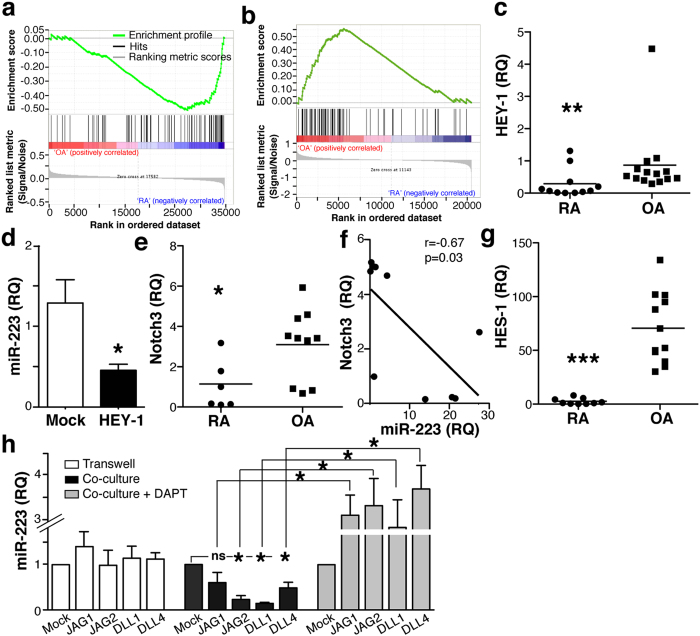
Notch activation downregulates miR-223 expression. (**a**) Enrichment plot (green) of GSEA using expression profiles of human macrophages isolated from SF of RA (*n* = 3) and synovial tissue of OA patients (n = 3). (**b**) GSEA as in *a* using expression profiles of human BMDC from RA (*n* = 9) and OA (*n* = 10) patients. The GSI_Notch gene set was used for GSEA (**a**,**b**). The bottom section shows the ranking metric value, which indicates gene correlation with a phenotype. (**c**) Relative HEY-1 mRNA levels in CD14^+^ cells from RA and OA patients. (**d**) miR-223 levels in HEK-293T cells overexpressing HEY-1 or an irrelevant cDNA (GFP; mock). (**e**) Determination of Notch3 mRNA levels in RA and OA isolated cells. (**f**) Linear regression analysis of Notch3 and miR-223 levels in RA and OA samples. Only samples with paired determinations of Notch3 and miR-223 were included in the analysis (*n* = 11). (**g**) Relative mRNA levels of the Notch effector HES-1 in the indicated samples. (**h**) Monocytes healthy donors were co-cultured with mock or Notch ligand-expressing OP-9 cells in conditions without (transwell) or with cell-cell contact (co-culture), alone or with the Notch inhibitor DAPT. After cell sorting, monocyte miR-223 levels were determined by RT-qPCR. For (**c**,**e**,**f**,**g**) each point represents one patient. For (**d**,**h**), data are mean ± SEM from triplicate RQ determinations in at least 3 independent experiments. *p < 0.05, **p < 0.01, Mann-Whitney *U*-test.

To study miR-223 regulation in RA macrophages, we searched the Ensembl database (v.77) for putative miR-223 promoter-binding transcription factors (TF). Scanning of the 10 Kb upstream of the miR-223 sequence identified two main regulatory regions in which TF binding sites were identified by the ENCODE Project (Supplementary Table S1). Of the TF predicted to bind the miR-223 promoter, we focused on the repressor HEY-1 (hairy/enhancer of split-related -HESR-with YRPW motif protein 1) for several reasons. First, at difference from Srf (serum response factor), Egr1 (early growth response) or TAF1 (TATA box binding protein-associated factor 1), all involved in multiple regulatory programs, HEY-1 is a very specific target of the Notch pathway. In addition, whereas PU.1 is a well-recognised myeloid transcription factor that could upregulate miR-223, Notch pathway and HEY-1 involvement in miR-223 regulation has not been studied in myeloid cells. Finally, study of gene expression profiles for human SF CD14^+^ cells from RA and tissue macrophages from OA patients (GSE49604) (ref. 32) using gene set enrichment analysis (GSEA) showed diminished Notch signalling in RA compared to OA cells (p < 0.05; Fig. 2a). GSEA also showed a tendency (FDR = 0.28; Fig. 2b) to enriched Notch signalling in human bone marrow-derived cells (BMDC) from OA compared to RA patients^33^ (GSE27390). Consistent with these analyses, HEY-1 expression was lower in RA than in OA CD14^+^ cells (Fig. 2c). In HEK-293T cells, HEY-1 overexpression led to significant reduction in miR-223 expression (Fig. 2d), which validated HEY-1 as a miR-223 transcriptional regulator.

Analysis of Notch isoforms showed similar Notch1 and Notch2 levels in OA and RA CD14^+^ cells (Supplementary Fig. S2). Notch3 mRNA was nonetheless lower in RA than in OA cells (Fig. 2e), and Notch3 mRNA and miR-223 levels showed negative correlation (p = 0.03, r = 0.68; Fig. 2f). Levels of HES-1, another Notch target, were lower in RA cells (Fig. 2g); negative correlation was also found between HES1 and miR-223 levels in our cohort (not shown), which supports GSEA data indicating that the Notch pathway is less active in RA than in OA macrophages.

To determine whether Notch signalling regulates miR-223 expression in myeloid cells, we co-cultured monocytes from healthy donors with OP-9 cells, a bone marrow-derived mesenchymal stem cell line that does not express Notch ligands, or with human Notch ligand-transduced OP-9 subclones; miR-223 was determined in monocytes isolated by cell sorting. OP-9 cells do not express macrophage colony-stimulating factor^34^, which precluded monocyte expansion and/or differentiation during co-culture.

Monocyte miR-223 expression was unaltered when cells were co-cultured with Notch ligand-expressing OP-9 cells in transwells (Fig. 2h). When cell-cell contact was allowed, OP-9 cells that expressed Jagged2 (JAG2), delta-like1 (DLL1) or delta-like4 (DLL4) induced significant monocyte miR-223 downregulation (Fig. 2h). JAG1-expressing OP-9 cells did not cause miR-223 repression in monocytes (p = 0.14, Mann-Whitney *U*-test).

Monocytes and OP-9 cells were also co-cultured with DAPT (N-[N-(3,5-difluorophenacetyl)-l-alanyl]-S-phenylglycine t-butyl ester), a gamma-secretase inhibitor that blocks Notch activation^35^. DAPT reversed the inhibitory effect of the Notch ligands on monocyte miR-223 levels (Fig. 2h). miR-223 expression was slightly although not significantly higher in monocytes co-cultured with DAPT-treated ligand-expressing OP-9 cells compared to OP-9 mock cells (Fig. 2h), suggesting basal Notch-mediated repression of miR-223 in monocytes. These results indicate that Notch signalling represses miR-223 in myeloid cells.

### *In silico* search for miR-223 targets

Although >20 genes have been identified as miR-223 targets in distinct cell types, only four are validated in monocytes/macrophages; these include the IKKα (inhibitor of NF-κB kinase α), the NLRP3 (NOD-like receptor family, pyrin domain containing 3) inflammasome, PKNOX1, and STAT3 (signal transducer and activator of transcription)^36^,^37^. miR-223-mediated silencing of NLRP-3, Pknox1, and STAT3 is counterintuitive based on the pro-inflammatory phenotype of RA macrophages. IKKα targeting by miR-223 relieves inhibition of the canonical NF-κB pathway, which enhances production of pro-inflammatory cytokines and chemokines^38^. We found no differences in *ikk*α mRNA (Supplementary Fig. S3a) and IKKα staining by immunohistochemistry (IHC) between RA and OA synovial tissues (Supplementary Fig. S3b).

**Figure 3:**
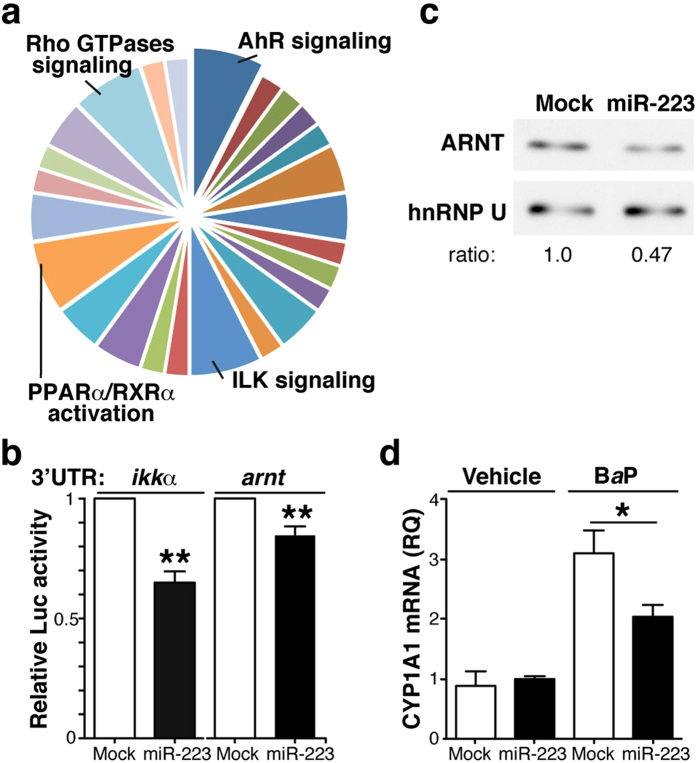
miR-223 target ARNT expression and signalling. (**a**) Ingenuity analysis of putative miR-223 targets as determined *in silico*. Signalling pathways with the highest scores are indicated. (**b**) Luciferase reporter assays of HEK-293 cells transduced with pre-miR-223 or a control (mock), and transfected with the wild-type *ikk*α- or the *arnt* 3′UTR reporter constructs. Luciferase activity was normalised to that of mock-transfected cells. Data shown as mean ± SEM of triplicates in one representative experiment (*n* = 3). (**c**) Immunoblot analysis of extracts of HEK-293 cells expressing control or pre-miR-223, probed with anti-ARNT and -hnRNP U (nuclear loading control); the hnRNP U/ARNT ratio in mock-transfected cells is equal to 1. For (**b**,**c**), data are representative of at least three experiments. (**d**) CYP1A1 mRNA levels in mock and pre-miR-223-expressing THP-1 cells treated with B*a*P or vehicle. Data shown as mean ± SEM of RQ from triplicates in three independent experiments. *p < 0.05, **p < 0.01, Mann-Whitney *U*-test.

We searched for unknown miR-223 targets using *in silico* enrichment analysis. Ingenuity pathway analysis (IPA) linked these predicted miR-223 targets to 25 signalling pathways (Supplementary Fig. S4), the most prominent of which were those of the AHR, integrin-linked kinase (ILK), PPAR-α/retinoid X receptor (RXR)-α, and small Rho GTPases (Fig. 3a). We focussed on the AHR pathway because of its relevance in RA pathology. miR-223 was predicted to interact with ARNT, a necessary partner for AHR-driven transcription.

We found 12 transcripts in Ensembl annotated for the human *arnt* gene, of which only six registered a 3′UTR region. FindTar predicted, with high reliability, four binding sites for miR-223-3p and seven for miR-223-5p in the *arnt* 3′UTR (Supplementary Table S2); most of these were located in regions of exact sequence identity among the isoforms, which could indicate redundant gene regulation by miR-223.

### Identification of ARNT as a miR-223 target

To determine whether the *arnt* 3′UTR is a miR-223 target, we transduced HEK-293T cells with pre-miR-223 or a mismatched miR (mock), and a cytomegalovirus promoter-driven luciferase construct fused to the *arnt* 3′UTR region. As a positive control, we used a similar luciferase reporter system under *ikk*α 3′UTR control, which miR-223 should target. Expression of the miR-223 mimic significantly reduced both *ikk*α and *arnt* 3′UTR-driven luciferase expression (Fig. 3b). The large number of miR-223 binding sites on the *arnt* 3’UTR precluded mutagenesis approaches to confirm silencing specificity. ARNT protein levels were reduced in pre-miR-223-expressing HEK-293T cells (Fig. 3c), which supports miR-223 targeting of *arnt* mRNA. In addition, pre-miR-223 expression in THP-1 myeloid cells attenuated B*a*P-induced upregulation of CYP1A1 (Fig. 3d), an AHR effector^10^. Since pre-miR-223 overexpression did not affect AHR protein levels in these cells (Supplementary Fig. S5), our results suggest that high miR-223 levels functionally impair the AHR/ARNT pathway in myeloid cells by reducing ARNT protein levels.

### miR-223 counteracts AHR/ARNT-mediated inhibition of pro-inflammatory cytokines

**Figure 4:**
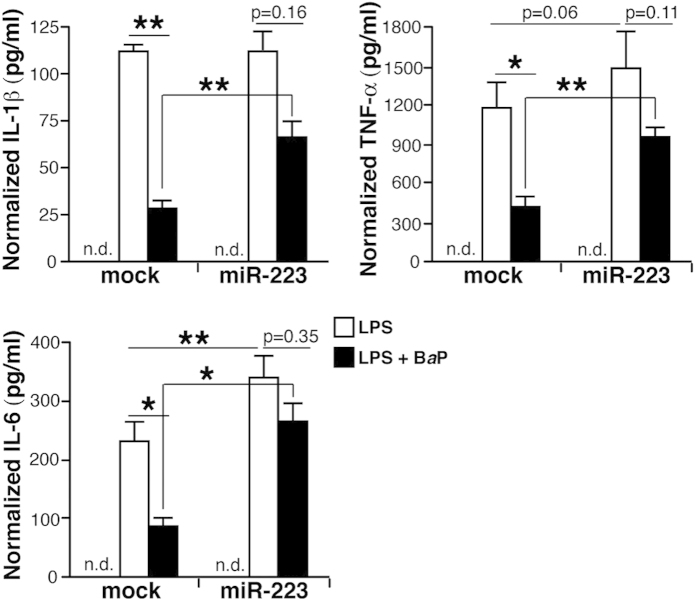
miR-223 reverses AHR/ARNT-induced inhibition of pro-inflammatory cytokines. ELISA analyses for IL-1β, TNF-α and IL-6 levels in the supernatant of mock- and pre-miR-223-transfected THP-1 cells stimulated with vehicle, LPS or LPS + B*a*P. Values are expressed as the concentration of cytokine (pg/ml) per mg cell extract. Data shown as mean ± SEM of values in three independent experiments. *p < 0.05, **p < 0.01 Student’s two-tailed *t*-test; n.d., not detected in vehicle-stimulated.

AHR agonists suppress LPS-induced expression of TNF-α, IL-6 and IL-1β^21^,^22^,^23^, which are important cytokines in RA aetiopathogenesis. We quantified the levels of these cytokines in control or pre-miR-223-expressing THP-1 cells exposed to LPS alone or with B*a*P. Pre-miR-223 expression tended to increase cytokine levels after LPS stimulation, which was significant only for IL-6 (Fig. 4). B*a*P significantly decreased LPS-induced cytokine expression in mock-transfected cells, but pre-miR-223 partially reversed B*a*P-induced inhibition (Fig. 4). Moreover, cytokine production was significantly higher in pre-miR-223-expressing THP-1 cells stimulated with LPS+B*a*P than in controls (Fig. 4). High miR-223 levels thus override AHR-mediated repression of pro-inflammatory cytokines in myeloid cells.

### The AHR/ARNT pathway is impaired in RA synovial tissue

**Figure 5:**
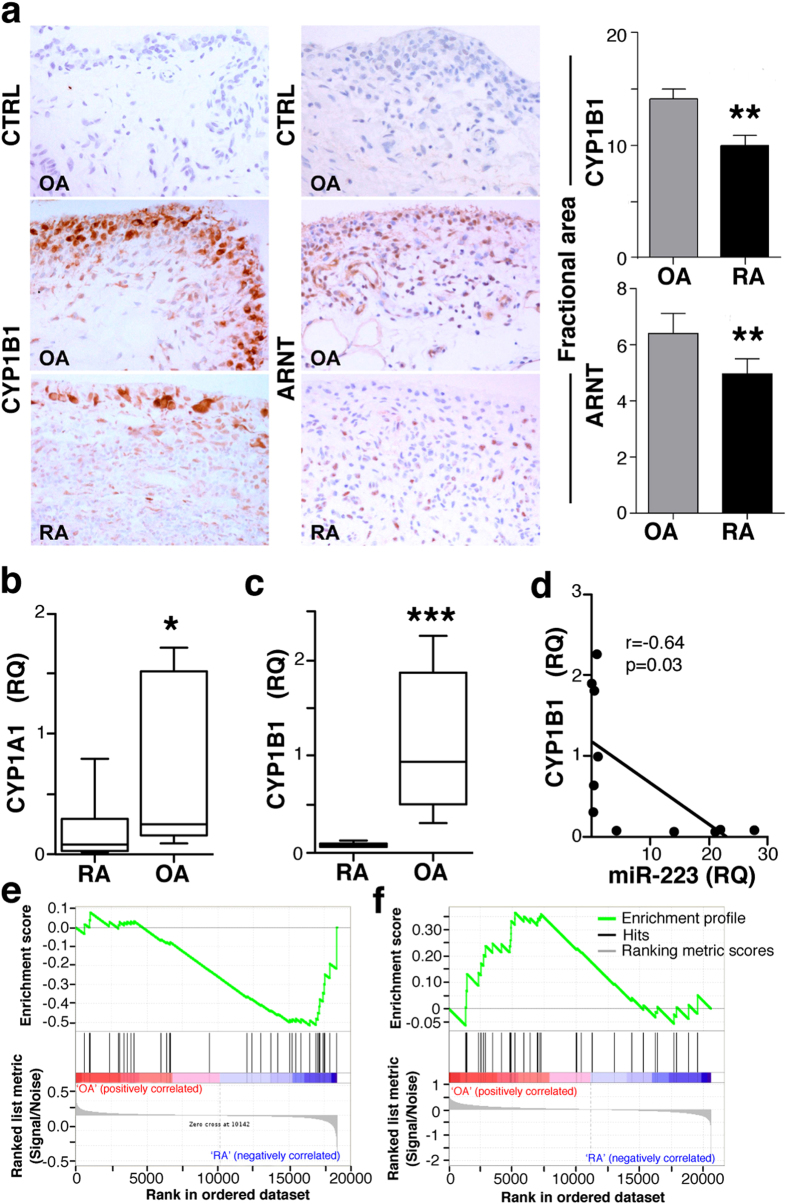
The AHR/ARNT pathway is downmodulated in RA compared to OA. (**a**) Immunohistochemical analysis of CYP1B1 (left) and ARNT (right) staining in RA (*n* = 13) and OA (*n* = 8) tissues. Staining with an irrelevant antibody is also shown (CTRL). Fractional immunostained area was quantified in digitalised images using ImageJ software. (**b,c**) CYP1A1 and CYP1B1 levels in cells isolated from RA (*n* = 6) and OA (*n* = 8) patients. RQ values were calculated from triplicates for each sample. (**d**) Linear regression analysis of CYP1B1 mRNA and miR-223 levels in RA and OA samples (*n* = 11). (**e**,**f**) Enrichment plot (green) of GSEA using expression profiles of human SF-derived macrophages (**e**) from RA (n = 6) and controls (n = 2), or BMDC (**f**) from RA (*n* = 9) and OA (*n* = 10) patients. The AHR gene set was obtained from PubMed (BIOGRID http://www.ncbi.nlm.nih.gov/gene/196). The bottom shows the ranking metric value, which indicates gene correlation with a phenotype.

We tested whether miR-223 levels are associated to impaired activation of the AHR/ARNT pathway in RA patients. In synovial tissues from OA and RA patients, we used IHC to analyse the CYP1B1 staining as a functional surrogate marker of AHR/ARNT pathway differences. Double immunofluorescent labelling showed variable CYP1B1 expression by CD68^+^ lining macrophages in OA and RA synovia, although the frequency of CD68^+^ cells with very low or undetectable CYP1B1 staining appeared to be higher in RA than in OA samples (Supplementary Fig. S6); CYP1B1 was also detected in other CD68^−^ cell types. IHC indicated profuse cytoplasmic CYP1B1 expression in fibroblastic and large mononuclear cells of lining and sublining areas in RA and OA synovial tissues (Fig. 5a); evaluation showed that the fractional area immunostained by CYP1B1 was larger in OA than in RA tissues (p = 0.002). This increased CYP1B1-stained area correlated with more ARNT^+^ cell accumulation in lining areas of OA than of RA tissues (p = 0.01; Fig. 5a). The data suggest attenuation of the AHR/ARNT pathway in RA compared to OA patients.

Analysis of AHR targets also indicated lower CYP1A1 (Fig. 5b) and CYP1B1 mRNA levels (Fig. 5c) in CD14^+^ cells isolated from RA than from OA patients; CYP1A2 was not expressed in CD14^+^ cells (not shown). We also found negative correlation in macrophage expression of miR-223 and CYP1A1 (p = 0.05; r = 0.60; Supplementary Fig S7a) and CYP1B1 (p = 0.03, r = 0.64; Fig. 5d), which suggests that miR-223 upregulation in RA CD14^+^ cells impairs AHR/ARNT-mediated transcription. We found no differences in AHR mRNA levels between OA and RA patients in our cohort (Supplementary Fig. S7b), which suggests that the functional impairment of the AHR/ARNT pathway is a result of reduced ARNT protein levels.

GSEA indicated a tendency to reduced AHR pathway signalling in RA macrophages (FDR = 0.1; GSE49604; Fig. 5e); the AHR gene set enrichment in OA cells was statistically significant when gene expression profiles for BMDC from RA and OA patients were analysed (FDR ≤0.25; Fig. 5f). These data reinforce the idea that the AHR/ARNT pathway is less active in RA than in OA patients.

### miR-223 prevent AHR/ARNT-mediated Notch3 upregulation

**Figure 6:**
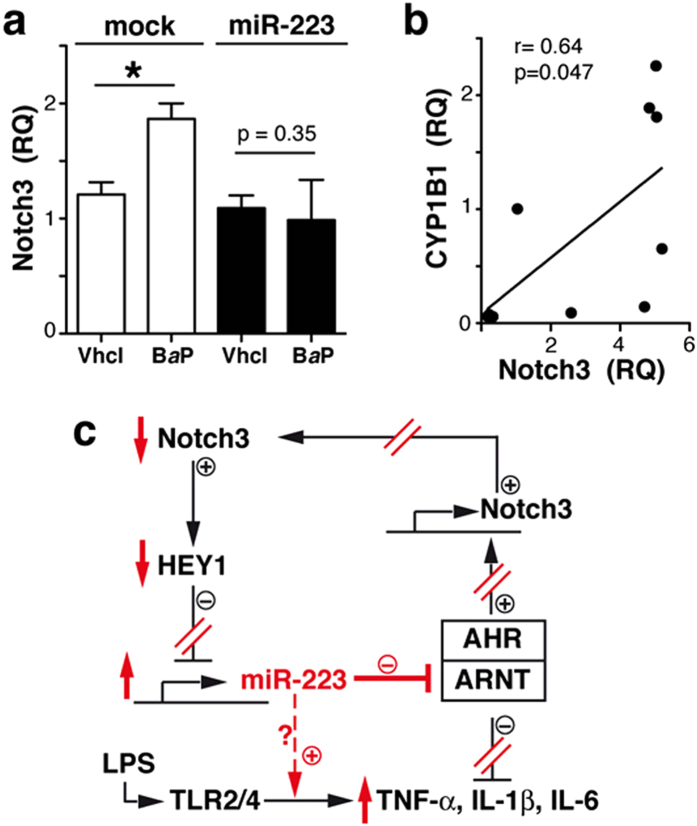
miR-223 prevents AHR/ARNT-induced Notch3 upregulation. (**a)** Notch3 mRNA levels in THP-1 cells expressing control (mock) or pre-miR-223 stimulated with B*a*P or vehicle. Data shown as mean ± SEM of RQ values (triplicates) from three independent experiments. (**b**) Linear regression analysis of CYP1B1 and Notch3 RQ values in RA and OA samples (*n* = 10). (**c**) Proposed model of miR-223 regulation in macrophages; miR-223 activity in RA macrophages indicated in red. *p < 0.05, **p < 0.01, ***p < 0.001, Mann-Whitney *U*-test.

AHR activation upregulates Notch expression in hepatic and intestinal innate immune cells^39^. We hypothesised that high miR-223 levels in RA cells could reverse its own inhibition by reducing Notch3. To analyse whether miR-223 influences Notch3 expression downstream of AHR in myeloid cells, control miR- or pre-miR-223-transfected THP-1 cells were stimulated with B*a*P. AHR activation increased Notch3 mRNA levels in THP-1 control cells, but not in those expressing pre-miR-223 (Fig. 6a). Our data suggest a regulatory network that involves Notch3, miR-223 and AHR. This is supported by the positive correlation between Notch3 and CYP1A1 (p = 0.04; r = 0.66; Supplementary Fig S7c) and CYP1B1 mRNA levels (p = 0.047, r = 0.64; Fig. 6b) in our cohort. We therefore propose a model (Fig. 6c) in which high miR-223 levels in RA macrophages impair the AHR/ARNT pathway, which reduces Notch3 expression and prevents the AHR/ARNT-induced inhibition of pro-inflammatory cytokines relevant in RA pathogenesis.

## Discussion

Here we combined gene expression and advanced bioinformatics analyses to demonstrate that (i) miR-223 is selectively upregulated in CD14^+^ cells from SF of RA patients with active disease; (ii) Notch activation represses miR-223 expression in myeloid cells; (iii) miR-223 targets ARNT, a necessary co-receptor for AHR-dependent transcriptional activity; (iv) miR-223 prevents AHR-mediated inhibition of pro-inflammatory cytokines; and (v) AHR target genes and ARNT protein itself are downmodulated in RA compared to OA synovial tissues. GSEA using gene expression profiles from two independent study^32^,^33^ using lesional SF macrophages and BMDC isolated from RA and OA patients, respectively, also detected reduced Notch and AHR signalling in RA-derived cells. Functional impairment of the AHR/ARNT pathway in RA patients might thus be a systemic phenomenon, not limited to lesional macrophages in our patient cohort.

In our analyses, we compared CD14^+^ cells isolated from synovial fluid (RA) and synovial tissue (OA). Despite their distinct sources, CD14^+^ cells isolated from SF and tissue should be considered comparable. There is no direct traffic from the bloodstream to the SF, and SF CD14^+^ cells thus derive from cells detached from the synovial lining layer. Morphological criteria indicate that SF CD14^+^ cells are not monocytes but macrophage-like cells^25^ with a genetic program resembling that of M1-skewed macrophages^26^. miR-223 levels decrease during monocyte-to-macrophage differentiation^38^, and this reduction leads to higher *ikk*α mRNA levels in macrophages than in monocytes. In our study, we found no difference in *ikk*α mRNA levels between RA-CD14^+^ cells from SF and OA-CD14^+^ cells from synovial tissue (Supplementary Fig. S3a), which reinforces the idea that CD14^+^ cells isolated from SF are not monocyte-enriched.

Studies of T cell acute lymphoblastic leukaemia showed that the Notch pathway can repress^40^ or induce^41^ miR-223 expression. Our results pinpoint Notch activation, and specifically HEY-1 as a direct repressor of miR-223 in myeloid cells. Reduced Notch signalling in RA macrophages, as evidenced by low HEY-1 and HES-1 levels, might affect miR-223 transcription directly (release of a brake in its promoter) or modify the activity of other miR-223 positive regulators such PU.1 (ref. 6). Indeed, Notch signalling restricts the extent of PU.1 activity in pro-T cells^42^. The mechanism responsible for impaired Notch signalling in our RA cells is nonetheless unclear. We detected lower Notch3 mRNA levels in RA SF macrophages, but Notch1 and Notch2 were unaltered relative to OA cells. Evidence indicates a non-redundant function of Notch ligands and receptors in cell function^43^,^44^. miR-223 repression might thus be a Notch3-mediated event induced by specific ligand interaction. In co-culture experiments, DLL1 and JAG2 inhibited miR-223 to a greater degree than DLL4; JAG1 had no effect. JAG2 induces the strongest Notch3-mediated signals in T cells of all the Notch ligands^44^, and provides anti-inflammatory signals in lung cancer cells^43^. Decreased Notch3 levels could also reduce Notch-mediated signalling strength in RA macrophages, leading to a specific differentiation pattern. Signalling strength is a major regulatory factor of Notch-mediated differentiation in human α/β or γ/δ T cells^44^, endothelial cell fate determination^45^ and of patterning in inner ear development^46^, in which Notch signalling strength is determined by competition between DLL1 and JAG1 ligands in adjacent cells. These examples show that combinatorial arrays of receptor-ligand cis- and trans-interactions could affect Notch signals, prompting distinct responses depending on cell or tissue context.

Here we link miR-223 to the AHR pathway, which is implicated in RA pathogenesis^47^. Although IPA suggested four putative miR-223 targets in the AHR pathway, we focussed on ARNT as a direct AHR regulator. Bioinformatics predicted that miR-223-3p and miR-223-5p both bind with imprecise base-pairing to multiple sites at the ARNT-3′UTR. This is consistent with the translational repression of ARNT, rather than mRNA degradation, observed after pre-miR-223 overexpression. The multiple miR-223 sites at the ARNT-3′UTR resemble other miR, including *let-4* (ref. 48), and are usually associated with enhanced specificity and translational suppression. miR-223 overexpression prevented AHR-mediated inhibition of IL-6, TNF-α and IL-1β production in LPS-stimulated myeloid cells, which functionally validates AHR pathway targeting by miR-223.

The reduction of ARNT protein levels and AHR target genes in RA compared to OA synovial tissues suggests a miR-223/ARNT connection for human disease. We propose a model in which elevated miR-223 levels in RA macrophages reduce ARNT translation and thus prevent AHR-driven immunosuppression in these cells (Fig. 6c). Moreover, miR-223 negatively regulates AHR/ARNT-mediated Notch3 expression and could contribute to release of Notch-mediated miR-223 repression, augmenting its levels. miR-223 might also interact via an AHR-independent, TLR-dependent mechanism to boost induction of pro-inflammatory cytokines, particularly IL-6, in myeloid cells. miR-223 upregulation could thus increase RA macrophage sensitivity to pro-inflammatory and reduce their responsiveness to anti-inflammatory signals.

Our results imply that, in RA macrophages, miR-223 is a central regulator of the AHR/ARNT pathway, which might be activated by endogenous (e.g., kynurenic acid) and/or exogenous (e.g., tobacco smoke) ligands that can influence RA aetiology. The AHR pathway leads to pro- and anti-RA activities; AHR activation could promote RA development by inducing Th17 differentiation^15^ and could simultaneously provide RA-inhibitory signals such as Treg cell differentiation^16^,^19^ or impaired macrophage activation^21^,^22^,^23^. Elevated miR-223 levels in RA macrophages would render these cells insensitive to AHR/ARNT-induced inhibition of the production of TNF-α, IL-6 and IL-1β, critical cytokines in RA pathology. Whether miR-223 targets the AHR pathway in other RA synovial inflammatory cells will require further research. For instance, miR-223 is expressed strongly in naïve T cells but is barely detectable in RA Th17 cells^27^, which might facilitate AHR-driven Th17 cell development. High miR-223 levels correlate with reduced Treg cell numbers in maternal and cord blood of pregnant women exposed to tobacco smoke^49^, suggesting that miR-223 expression impairs AHR-induced Treg cell differentiation.

In summary, we demonstrate that high miR-223 expression in myeloid cells prevents AHR-induced repression of TNF-α, IL-6 and IL-1β. This miR-223/AHR pathway connection provides conceptual support for miR-223 antagonist-based therapies in RA^28^, and identifies a means to fine-tune AHR activity in myeloid cells.

## Methods

### Patients and synovial tissues

For microarray and validation studies, macrophages were obtained by centrifugation on Ficoll-Paque (GE-Healthcare) of SF from RA patients with active knee arthritis, or by collagenase I digestion of synovial tissue from OA patients who had undergone knee joint replacement surgery; both procedures were followed by magnetic separation of CD14^+^ cells (Miltenyi Biotec). The mean age of RA patients was 63 ± 5 years, and 73% were female; the mean age of OA patients was 73 ± 6 years, and 57% were female. Three patients received only non-steroid anti-inflammatory drug therapy, and four received disease-modifying anti-rheumatic drugs (3 methotrexate and one hydroxychloroquine) plus low dose steroids. The clinical characteristics of RA patients (*n* = 11) used for miR validation are shown in Supplementary Table S3. None of the RA or OA patients in the microarray and validation cohorts were active smokers at the time of sample collection.

Synovial tissues for IHC studies were derived from arthroscopic knee biopsy of RA patients with active arthritis (*n* = 13) or by surgical synovectomy of OA patients (*n* = 8) who underwent knee joint replacement surgery. Tissues were formalin-fixed and paraffin-embedded. Patients gave informed consent and the study was approved by the ethics committees of the Hospital 12 de Octubre (Madrid, Spain) and the Hospital Clinic (Barcelona, Spain). The methods were carried out in accordance with the approved guidelines.

### RNA isolation and microarray analyses

RNA was extracted using Trizol reagent (Invitrogen), and integrity assessed in a Bionalyzer 2100 (Agilent). Total RNA (1 μg) from three RA and OA samples was labelled with miRCURY LNA microRNA Hy3/Hy5 Power labelling kit and hybridised onto slides in the human miRCURY LNA microRNA Array Kit v.11.0 (Exiqon). Slides were scanned at 5 μm resolution in an Axon 4000B scanner (Molecular Devices), and spots quantified with GenePix 5.0 software. Raw data (deposited in GEO; http://www.ncbi.nlm.nih.gov/geo/query/acc.cgi?token=ipilmwaerdujfwr&acc=GSE63745) were normalised and differential expression analysed using the Bioconductor Limma software. FDR was used to correct p-values.

### Cell culture

HEK 293T cells (ATCC) were cultured in Dulbecco’s Modified Eagle Medium (DMEM; BioWest, Nuaillé, France) supplemented with 10% foetal calf serum (FCS), 1 mM sodium pyruvate and 2 mM L-glutamine. THP-1 cells (ATCC) were cultured in RPMI 1640 (BioWest) supplemented with 10% FCS, 2 mM L-glutamine, 1% non-essential amino acids and 0.05 mM 2-mercaptoethanol. OP-9 cells and subclones (see below) were cultured in minimum essential medium (MEM)-α (Life Technologies) supplemented with 20% FCS, 1 mM sodium pyruvate, 2 mM L-glutamine and 3 mM sodium bicarbonate. All media also contained penicillin/streptomycin (100 U/mL). All additives were from Sigma-Aldrich.

### Transfection of HEK-293T cells and luciferase assay

HEK-293T cells were transfected with human HEY-1 (provided by J.L. de la Pompa; Centro Nacional de Investigaciones Cardiovasculares, Madrid, Spain) using jetPEI (Polyplus), or with pLV-hsa-mir-223 and pLV-mir-control vectors (Biosettia) using the CaCl_2_ method. Cells were selected with puromycin (3 μg/ml) and transfected with miTarget miRNA Target Sequence (3′UTR) Expression Clones for *arnt* and *ikk*α (Labomics). Reporter gene expression was analysed with the Dual-Luciferase Reporter Assay System (Promega). ARNT protein levels were analysed in nuclear extracts using anti-ARNT (clone D28F3; Cell Signaling) and anti-hnRNPU antibody (loading control; Abcam). AHR protein levels were analysed in total cell extracts using anti-AHR (clone RPT1; Thermo Fisher) and anti-β-actin (clone AC-15, Sigma-Aldrich).

### Transduction of OP-9 cells with Notch ligands

pLZRS-IRES-eGFP retroviral constructs, empty or encoding human Delta-like1 (DLL1) or Jagged1 (JAG1), were provided by L. Parreira (Instituto de Histologia e Embriologia, Lisbon, Portugal). Human Jagged2 (JAG2) cDNA, PCR-amplified from total RNA isolated from HeLa cells, and human Delta-like4 (DLL4) full-length cDNA (provided by G. Tosato, National Institutes of Health, Bethesda, MD, USA) were subcloned into pLZRS-IRES-eGFP. Individual retroviral constructs were transfected into the packaging 293T Phoenix-Amphotropic cell line (provided by G. Nolan, Stanford University, Stanford, CA, USA), and supernatants obtained from puromycin-selected transfected cells were used to transduce OP-9 cells (ATCC) by centrifugation in the presence of polybrene. Transduced OP-9 cells with equivalent GFP expression levels were isolated by cell sorting (FACSVAntage) 48 h post-transduction.

### Co-culture of monocytes with OP-9 cells

Purified CD14^+^ cells (>95%) from healthy donors were added (1 h, 37 °C) to mock- or Notch ligand-expressing OP-9 cells, in the presence of DMSO (vehicle) or DAPT (Merck Millipore); monocytes were isolated by cell sorting (MoFlo XDP; Beckman Coulter) based on lack of GFP expression. CD14^+^ cells were also plated in transwells (0.4 μm pore size; Costar) to prevent physical interaction with OP-9 cells during co-culture.

### Transfection and stimulation of THP-1 cells

THP-1 cells were transfected with the pre-miR-223 (10 μM; Life Technologies), or with a fluorescently-labeled negative control (5 μM) using Amaxa I and the Monocyte Nucleofector Kit (Lonza). Transfected THP-1 cells (~95% based on fluorescence labelling) were stimulated (1–3 h, 37 °C) with B*a*P (5 μM) or vehicle, followed by LPS (1–8 h, 37 °C). Cytokine levels were determined in supernatants using specific ELISA kits (BioLegend). Concentration was divided by total protein level in cell extracts to correct for cell number in each condition.

### qRT-PCR analyses

Total RNA was extracted from CD14^+^ cells isolated from RA or OA patients, or from cultured cells using TRI Reagent (Sigma-Aldrich). For miR qRT-PCR, retrotranscribed RNA (25 ng) was mixed with specific primers (TaqMan MicroRNA Reverse Transcription Kit; Life Technologies) in an ABI PRISM 7900 HT (Applied Biosystems) using a TaqMan MicroRNA-based system. For quantitative mRNA analyses, total RNA (1 μg) was retrotranscribed with the High Capacity Reverse transcription Kit (Life Technologies) using random primers, and then amplified with the primers listed (Supplementary Table S4) using a Hot FIREPol EvaGreen Plus-based system. The relative quantity (2^−ΔΔCt^) of each miR or mRNA was calculated using as reference the sample with the highest C_t_ value after normalisation to U6 Taqman probe (for miR analyses) or β-actin (for mRNA analyses).

### Immunolabelling of synovial tissue

For IHC staining, we used a standard indirect avidin-biotin peroxidase method (ABC standard; Vector Laboratories) and developed with diaminobenzidine. Samples were stained with anti-IKKα (ab47453), -CYP1B1 or -ARNT antibodies. Paraffin sections were pretreated by microwave heating in citrate (pH 6.0) or Tris-EDTA buffer (pH 9.0) for CYP1B1 and ARNT detection, respectively. Negative controls were incubated with non-immune IgG. CYP1B1/CD68 double-labelled samples were analysed by simultaneous CYP1B1 immunoperoxidase and CD68 immunofluorescent detection and an Alexa594 goat anti-mouse secondary antibody (Molecular Probes).

Synovial tissue sections were photographed and digitalised using an AxioCam ERc 5S camera and ZEN lite 2012 software on a Zeiss Scope. A1 microscope (Zeiss). Fractional immunostained area was quantified in digitalised images using ImageJ software (http://rsb.info.nih.gov/ij). Data were analysed using Prism software v6.0 (GraphPad). Quantitative variables were compared by the Mann Whitney *U*-test; a p value <0.05 was considered significant.

### GSEA analyses

Raw microarray data corresponding to BMDC from 10 OA and 9 RA patients were obtained from a public database^33^. Data were normalised using the RMA (quantile) approach and subjected to GSEA (http://www.broadinstitute.org/gsea/downloads.jsp), permutating 1000 times by phenotype. Illumina expression data from the GSE49604 study^32^ were re-analysed using quantiles normalisation and subjected to GSEA by permutating 1000 times by geneset. Enrichment in both cases was determined using gene sets of Notch (PMID: 19907488) and AHR.

### Bioinformatic analyses for identification of miR-223 targets

The sequences of the human 3′-UTR *arnt* gene, of mature microRNA hsa-miR-223-3p and hsa-miR-223-5p were retrieved from the Ensembl database (release 72, June 2013) and the miRBase (release 19, August 2012). Of the 12 transcripts annotated for the human *arnt* gene, only 6 for which the 3′-UTR is registered were used in the calculations. Predictions were made using the algorithms miRanda, PITA, FindTar v3.11.12, RNAHybrid, and TargetScan v6 using parameter default values. The sequences of six, seven and eight nucleotides of each miRNA 5′ end were considered as seed, starting from the first, second or third nucleotide. Mismatches were not allowed in the seed. Multiple sequence alignments were performed using ClustalW v2.1.

## Additional Information

**How to cite this article**: Ogando, J. *et al*. Notch-regulated miR-223 targets the aryl hydrocarbon receptor pathway and increases cytokine production in macrophages from rheumatoid arthritis patients. *Sci. Rep*. **6**, 20223; doi: 10.1038/srep20223 (2016).

## Supplementary Material

Supplementary Information
