# Supplementary material for: Notch-regulated miR-223 targets the aryl hydrocarbon receptor pathway and increases cytokine production in macrophages from rheumatoid arthritis patients
Source: Sci Rep. 2016 Feb 3;6:20223. doi: 10.1038/srep20223 (PMC4738320; doi:10.1038/srep20223)
Supplement: Supplementary Information [file srep20223-s1.pdf]

Notch-regulated miR-223 targets the aryl hydrocarbon receptor pathway and increases cytokine production in macrophages from rheumatoid arthritis patients

Jesús Ogando, Manuel Tardáguila, Andrea Díaz-Alderete, Alicia Usategui, Vanessa Miranda-Ramos, Dannys Jorge Martínez-Herrera, Lorena de la Fuente María J. García-León, María C. Moreno, Sara Escudero, Juan D. Cañete, María L. Toribio, Ildefonso Cases, Alberto Pascual-Montano, José Luis Pablos, Santos Mañes

The following supplementary material may be found in the online version of this article:

Table S1. Transcription factors predicted to bind to the miR-223 promoter

Table S2. Most probable sites predicted for hsa-miR-223-3p and hsa-miR-223-5p on the human *arnt* gene

Table S3. Clinical characteristics of RA patients

Table S4. List of primers used in the study

Figure S1. Non-validated miR expressed in RA and OA patient macrophages

Figure S2. Notch1 and Notch2 are not differentially expressed in RA and OA macrophages

Figure S3. IKKα is not differentially expressed in RA and OA macrophages

Figure S4. Ingenuity Pathway Analysis of miR-223 targets identified in silico

Figure S5. Forced miR-223 expression does not affect AHR protein levels

Figure S6. AHR target gene expression in synovial macrophages

Figure S7. Correlation between CYP1A1, miR-223 and Notch3 levels

Table S1. Transcription factors predicted to bind to the miR-223 promoter

Transcription factor	Location	Cell type
SRF	66017174-66017482	GM12878
PU1	66018575-66019006	GM12878
EGR1	66009236-66009546	K562
TAF1	66015066-66015773	K562
HEY-1	66015169-66019731	K562
PU1	66018574-66018961	K562

Table S2. Most probable sites predicted for hsa-miR-223-3p and hsa-miR-223-5p on the human *arnt* gene

<table>
<tr><th>miRNA</th><th>Ensembl transcript ID</th><th>Seedmatch details
(length/# wobbles/first nt)</th><th>Position in 3’-UTR</th><th>Prediction algorithm</th></tr>
<tr><td rowspan="5">hsa-miR-223-3p</td><td>ENST00000354396</td><td rowspan="3">6mer/0/3</td><td rowspan="2">1141-1146</td><td rowspan="12">FindTar</td></tr>
<tr><td>ENST00000358595</td></tr>
<tr><td>ENST00000471844</td><td>1526-1531</td></tr>
<tr><td rowspan="2">ENST00000504358</td><td rowspan="2">6mer/1/3</td><td>79-84</td></tr>
<tr><td>243-248</td></tr>
<tr><td rowspan="7">hsa-miR-223-5p</td><td>ENST00000354396</td><td rowspan="3">7mer/1/2</td><td rowspan="2">761-767</td></tr>
<tr><td>ENST00000358595</td></tr>
<tr><td>ENST00000471844</td><td>1146-1152</td></tr>
<tr><td>ENST00000354396</td><td rowspan="3">8mer/1/3</td><td rowspan="2">778-785</td></tr>
<tr><td>ENST00000358595</td></tr>
<tr><td>ENST00000471844</td><td>1163-1170</td></tr>
<tr><td>ENST00000358595</td><td>6mer/1/1</td><td>2096-2101</td></tr>
</table>

Site position is counted from the beginning of the 3'UTR. Sites conserved in different 3'UTRs were obtained by a multiple sequence alignment using ClustalW v2.1.

Table S3. Clinical characteristics of RA patients

Characteristic	number (% or SD)
Female	8 (73)
Age (SD)	63 (5)
Time of evolution, years (SD)	11 (8)
EROSIONS	8 (73)
RF+	7 (63)
ACPA+	8 (73)
Prednisone	4 (36)
DMARDs	5 (45)*

RF, Rheumatoid factor; ACPA, anti-citrullinated peptide antigen; DMARD, Disease modifying anti-rheumatic drugs; (*) 4 Methotrexate, 1 anti-malarials. No patients were on biologic therapy.

Table S4. List of primers used in the study

Gene	Forward	Reverse
b-act	CCCAGCACAATGAAGATCAA	CCCAGCACAATGAAGATCAA
ikkα	TGTGCCTCTTCTAGCAATGGA	TTCTGGTTTGTTGAGCAGCTT
hey1	CGAGCTGGACGAGACCAT	CTAGAGCCGAACTCAAGTTTCC
hes1	GTGAAGCACCTCCGGAAC	GTCACCTCGTTCATGCACTC
cyp1a1	CCCAGGCTCCAAGAGTCCACCC	GCCAGAAGAAACTCCGTGGCCG
cyp1b1	ACATCTTCGGCGCCAGCCAGG	TCCCTCCCCACGACCTGATCCA
il-6	CGGGAACGAAAGAGAAGCTCTA	GGCGCTTGTGGAGAAGGAG
il-1β	CCCTAAACAGATGAAGTGCTCCTT	GTAGCTGGATGCCGCCAT
notch1	TACAAGTGCAACTGCCTGCT	GGCAGACACAGGAGAAGCTC
notch2	TCAGCCGGGATACCTATGAG	TTTGCACAGGGATGAGACAG
notch3	CGTGGCTTCTTTCTACTGTGC	CGTTCACCGGATTTGTGTC
jag1	GTCCTAAGCATGGGTCTTGC	GGGTGTGGGATGCACTTATC
jag2	GTCGTCATCCCCTTCCAGT	GGTGGTATCGTTGTCCCAGT
dll1	GAGCGTGGGGAGAAAGTGT	ATGCTGCTCATCACATCCAG
dll4	GCACTCCCTGGCAATGTACT	GGAGTGGTGGGTGCAGTAGT

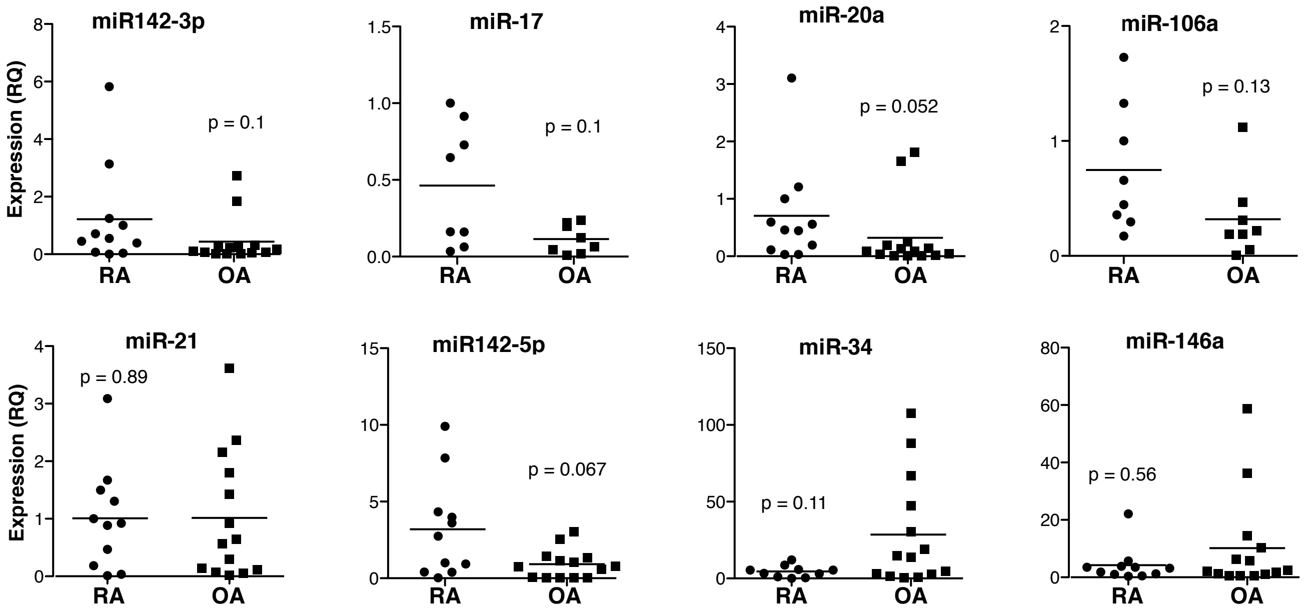

Figure S1. **Non-validated miR expressed in RA and OA patient macrophages.** Relative levels of the indicated miR were determined by RT-qPCR in macrophages isolated from RA (n = 11) and OA (n = 14) patients. Values are indicated as relative quantification (RQ), using the sample with the lowest $2^{-\Delta\Delta Ct}$ value (RQ = 1) as reference. Probability values were calculated using the Mann-Whitney U-test.

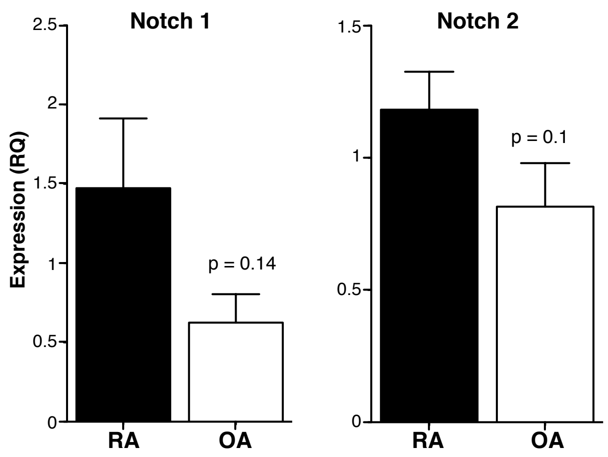

Figure S2. Notch1 and Notch2 are not differentially expressed in RA and OA macrophages. Relative levels of Notch1 and Notch2 mRNA in macrophages from RA (n = 4) and OA (n = 6) patients, as determined by RT-qPCR. Data shown as mean ± SEM; p values were calculated using the Mann-Whitney U-test.

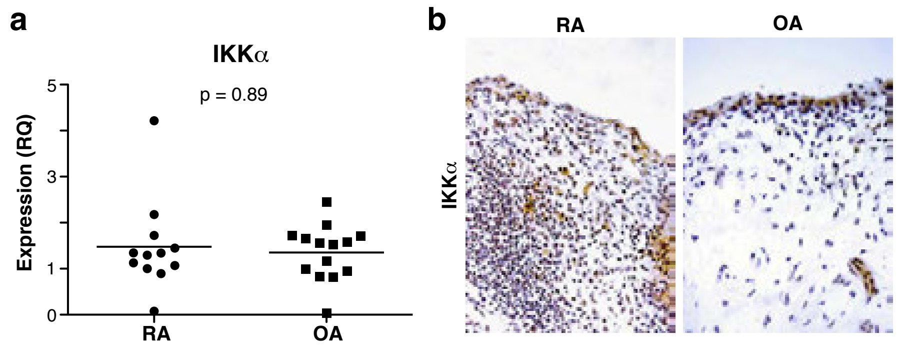

Figure S3. IKKα is not differentially expressed in RA and OA macrophages. (a) Relative IKKα mRNA levels in macrophages from RA (n = 12) and OA (n = 14) patients; Mann-Whitney U-test. **(b)** IKKα staining in RA (n = 13) and OA (n = 8) synovial tissues.

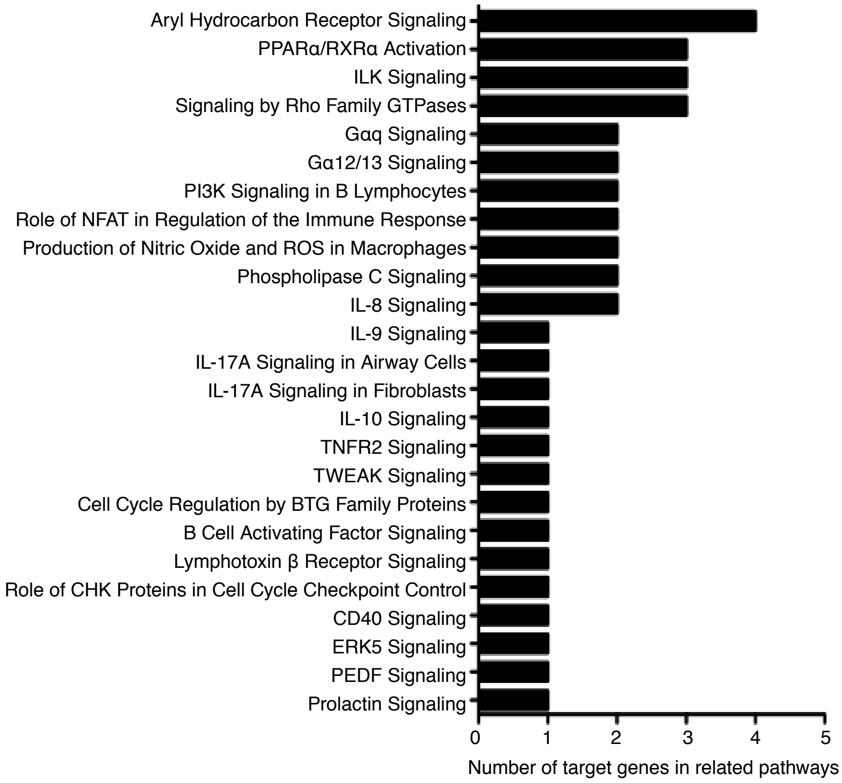

Figure S4. Ingenuity Pathway Analysis of miR-223 targets identified in silico. Number of putative miR-223 target genes for each of the signalling pathways identified by IPA.

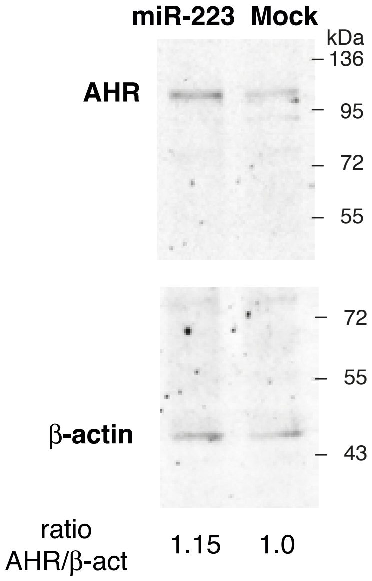

Figure S5. Forced miR-223 expression does not affect AHR protein levels. Immunoblot analysis of extracts of HEK-293 cells expressing control or pre-miR-223, probed with anti-AHR and -β-actin antibodies. Bands were densitometred in Image J and the AHR/β-actin calculared; ratio in mock-transfected cells is equal to 1. Results are representative of two independent experiments

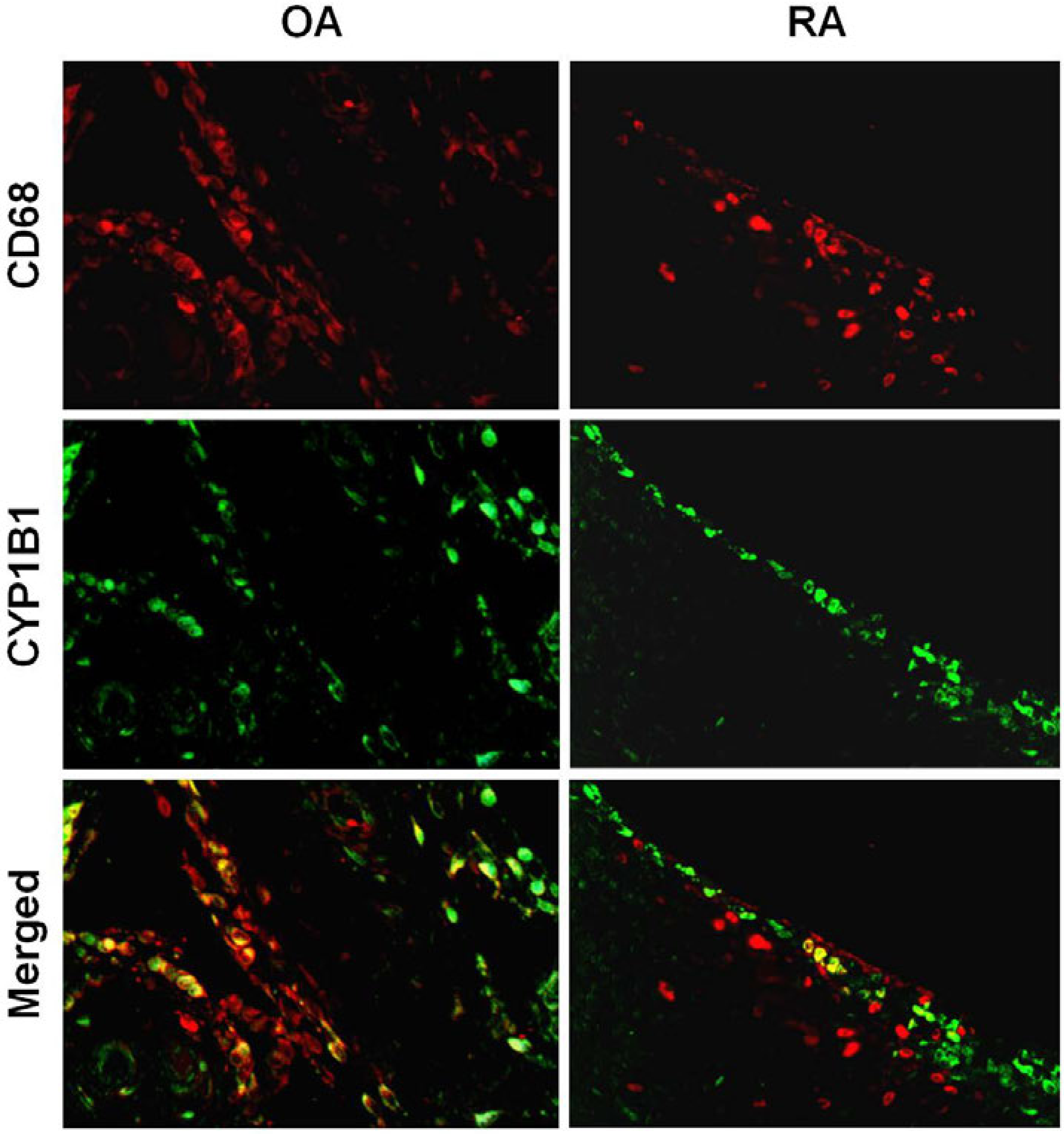

Figure S6. AHR target gene expression in synovial macrophages. Tissue sections from OA and RA patients were double-stained for CYP1B1 (green) and the macrophage marker CD68 (red); merge of both channels (yellow). Images representative of 5 synovial tissues per condition.

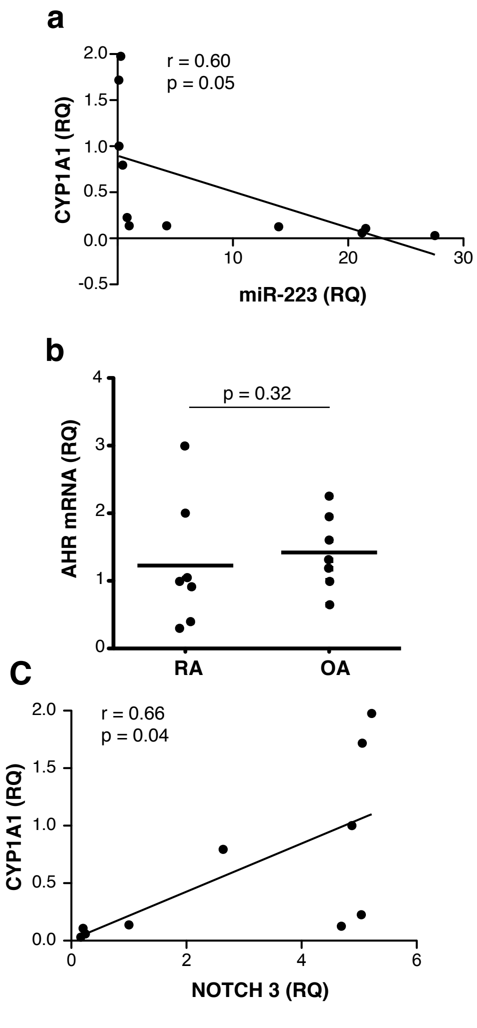

Figure S7. Correlation between CYP1A1, miR-223 and Notch3 levels. **(a)** Linear regression analysis of CYP1B1 mRNA and miR-223 levels in RA and OA samples (n = 11). **(b)** AHR mRNA levels in OA and RA patient samples, as determined by RT-qPCR. **(c)** Linear regression analysis of CYP1B1 and Notch3 mRNA levels in RA and OA samples (n = 10).
